# Cobalamin Related Parameters and Disease Patterns in Patients with Increased Serum Cobalamin Levels

**DOI:** 10.1371/journal.pone.0045979

**Published:** 2012-09-21

**Authors:** Johan F. B. Arendt, Ebba Nexo

**Affiliations:** Department of Clinical Biochemistry, Aarhus University Hospital, Aarhus, Denmark; Lund University Hospital, Sweden

## Abstract

**Background:**

Measurement of serum cobalamin levels is routinely used to diagnose cobalamin deficiency. Surprisingly, approximately 15% of patients have high cobalamin levels and no consensus exists regarding the clinical implications.

**Methods:**

Hospital-treated patients above 18 years of age referred for serum cobalamin measurement were included in groups of patients [percentage cobalamin supplemented] with low (<200 pmol/L, n = 200 [6%]), normal (200–600, n = 202 [6%]) high (601–1000, n = 217 [27%]) and very high (>1000, n = 199 [53%]) cobalamin levels. Total and cobalamin-saturated (holo) transcobalamin, total haptocorrin, soluble TC receptor, sCD320, and methylmalonic acid were analyzed. Data on diagnoses and medical prescriptions was obtained through medical files and the Aarhus University Prescription Database.

**Results:**

Among patients not cobalamin supplemented median total haptocorrin and holo transcobalamin levels were markedly higher in the groups with high/very high cobalamin levels compared to groups with low/normal cobalamin levels. Median total transcobalamin and sCD320 levels were similar across the groups. A number of diagnoses were significantly associated to very high Cbl levels (odds ratio (95% confidence interval)): alcoholism (5.74 (2.76–11.96)), liver disease (8.53 (3.59–20.23)), and cancer (5.48 (2.85–10.55)). Elevated haptocorrin levels were seen in patients with alcoholism, cancer, liver-, renal-, autoimmune-, and bronchopulmonary disease. No clinical associations to sCD320 and total and holo transcobalamin levels were found.

**Conclusion:**

In non-supplemented patients, high cobalamin levels were associated to high haptocorrin levels, and several diagnoses, including alcoholism, liver disease and cancer. Our study emphasizes that clinicians should take high serum cobalamin levels into consideration in the diagnostic process.

## Introduction

Measurement of serum cobalamin (vitamin B12, Cbl) is routinely used to diagnose or rule out a suspected Cbl deficiency. Therefore, we expect to obtain either a low or a normal value for serum Cbl. Surprisingly, a high fraction of patients display Cbl levels well above the upper limit of the reference interval [1], [2]. In our laboratory, we find that almost 15% of the patients referred for Cbl measurement have values above the reference range of 200–600 pmol/L (271–813 pg/mL), while only about 11% have values below the reference range (data not shown). The possible underlying causes for these findings are unclear, as are their clinical relevance.

Elevated serum Cbl levels are most consistently found in some types of myeloproliferative disorders, such as chronic myeloid leukemia, polycythemia vera, and hypereosinophilic syndrome. This is due to increased concentrations of haptocorrin (HC), one of the two circulating Cbl binding proteins [3]–[7]. Several studies have been conducted to link a number of other diseases or group of diseases to high Cbl levels and/or high levels of Cbl binding proteins [1]; [2]; [8]–[18]. These include different malignancies and hepatic-, renal-, infectious-, and autoimmune diseases. However, the studies are limited by a relatively small sample size and must be interpreted with caution. Moreover, the underlying alterations in Cbl related markers are not fully understood. Consequently, a lack of consensus exists regarding the clinical assessment of high serum Cbl levels.

This study presents a systematic evaluation of Cbl related parameters and the disease patterns in patients for whom routine measurements of serum Cbl were requested. We report that high Cbl levels are associated to an increased level of haptocorrin and to a number of diseases, most notably alcoholism, liver disease and cancer.

## Materials and Methods

### Blood Sample Collection and Patients

All patient serum samples were obtained as part of routine analysis on hospitalized patients, and only surplus serum was used for this study. Informed consent was not obtained from the patients, as approved by the Regional Ethics Committee of Central Jutland, Denmark (record nr.: 20090187). The committee reviewed and approved the entire study protocol, specifically the consent procedure. The study was approved by the Danish Data Protection Agency (record nr.: 2010-331-0378 and 2010-41-4559).

**Table 1:** Age and gender distribution of study population.

		Groups according to serum cobalamin			
Patients not in cobalamin supplementation therapy					
	Low (n = 189)	Normal (n = 190)	High (n = 159)	Very high (n = 94)	
**Age, mean (95% CI)**	54 (51–56)	58 (55–60)	56 (53–59)	62 (59–66)	*p = 0.003
**Sex, male (%)**	67 (35%)	104 (55%)	73 (46%)	42 (45%)	
**Patients in cobalamin supplementation therapy**					
	**Low (n = 11)**	**Normal (n = 12)**	**High (n = 58)**	**Very high (n = 105)**	
**Age, mean (95% CI)**	48 (37–60)	47 (35–59)	65(60–70)	68 (64–72)	*p = 0.0003
**Sex, male (%)**	5 (45%)	2 (17%)	19 (33%)	32 (30%)	

Basic characteristics of patients referred for measurement of serum Cbl levels and included in the study (n = 818). Age is displayed with means and corresponding 95% confidence intervals. Sex is displayed as number and fractions (%) of males.

* P-values were obtained by one-way analysis of variance when testing for difference in mean age across Cbl levels groups. Cbl: cobalamin, vitamin B12; 95% CI: 95% confidence interval.

Blood sample collection was conducted between 1^st^ of November 2009 and 31^st^ of December 2010 at the Department of Clinical Biochemistry, Aarhus University Hospital. Aarhus University Hospital covers all major medical fields in a region of approximately 400,000 inhabitants. During the study period of 14 months the laboratory performed 40,047 serum Cbl measurements. Approximately 30% were from hospital-treated patients and the remaining were from general practitioner (GP) treated patients.

**Table 2:** Cobalamin related parameters.

		Groups according to serum cobalamin			
	Low	Normal	High	Very high	
	**n_non-supplemented_** ** = 189**	**n_non-supplemented_** ** = 190**	**n_non-supplemented_** ** = 159**	**n_non-supplemented_** ** = 94**	
**Cobalamin related parameters**	**n_supplemented_ = 11**	**n_supplemented_ = 12**	**n_supplemented_ = 58**	**n_supplemented_ = 105**	
**Total serum cobalamin, pmol/L [200–600]**					
**- Not Cbl supplemented**	170 (149–186)	329 (262–429)	716 (652–830)	1328 (1165–2113)	
**- Cbl supplemented**	179 (148–181)	414 (258–484)	757 (673–884)	1358 (1129–2420)	
**Total transcobalamin, pmol/L [600–1,500]**					
**- Not Cbl supplemented**	970 (800–1140)	960 (840–1180)	1070 (820–1320)	1100 (880–1420)	p = 0.0002
**- Cbl supplemented**	1250 (1090–1400)	920 (780–1090)	980 (820–1380)	1300 (940–1880)	p = 0.005
**Holo transcobalamin, pmol/L [40–150]**					
**- Not Cbl supplemented**	41 (31–52)	64 (46–96)	150 (100–220)	180 (77–410)	p<0.0001
**- Cbl supplemented**	57 (44–66)	94 (79–116)	207 (145–290)	720 (370–1130)	p<0.0001
**Total haptocorrin, pmol/L [240–680]**					
**- Not Cbl supplemented**	510 (400–610)	620 (490–760)	730 (600–920)	1300 (900–2200)	p<0.0001
**- Cbl supplemented**	480 (370–680)	500 (450–550)	590 (490–770)	680 (550–840)	p = 0.0005
**Serum sCD320, arb.u. [12–97]**					
**- Not Cbl supplemented**	17 (14–21)	18 (16–22)	21 (17–33)	24 (19–36)	p<0.0001
**- Cbl supplemented**	20 (16–29)	18 (15–21)	23 (18–29)	32 (24–43)	p<0.0001
**MMA** a **, µmol/L [<0.28]**					
**- Not Cbl supplemented**	0.24 (0.19–0.34)	0.19 (0.15–0.27)	0.16 (0.13–0.20)	0.17 (0.13–0.24)	p<0.0001
	n = 155	n = 160	n = 120	n = 68	
**Impaired cobalamin status in patients with Cbl >250 pmol/L** b	–	41 (33%)	7 (6%)	14 (21%)	
	–	n = 124	n = 120	n = 68	

Medians (interquartile ranges) [reference ranges] are shown for Cbl related parameters in patients referred for measurement of serum Cbl divided according to Cbl supplementation (n = 818).

a Only non-supplemented patients with normal kidney function (eGFR ≥60 mL/min/1.73 m^2^, n = 503).

b Numbers and percentages of non-supplemented patients with normal kidney function and serum Cbl >250 pmol/L [31] showing biochemical Cbl deficiency, defined as holoTC <40 pmol/L and/or MMA >0.28 µmol/L. P-values were obtained by comparing medians across Cbl groups using Kruskal-Wallis test. Abbreviations: Cbl: cobalamin, vitamin B12; holo transcobalamin: Cbl-saturated transcobalamin; sCD320: soluble transcobalamin receptor CD320; MMA: methylmalonic acid; eGFR: estimated glomerular filtration rate.

Exclusion criteria were the following: patients <18 years of age or incomplete medical files (see below for details on clinical data) and a surplus blood sample containing <1 mL serum. The latter criteria led to exclusion of a large number of patients. Samples requested from GPs or private hospitals/clinics were excluded because medical files were not available.

The samples were divided into four groups according to Cbl levels: low: 55–199 pmol/L normal: 200–600 pmol/L (reference range in the local routine laboratory), high: 601–1000 pmol/L and very high: >1000 pmol/L. A priori, we decided to stop the collection when each of these groups contained approximately 200.

**Figure 1:**
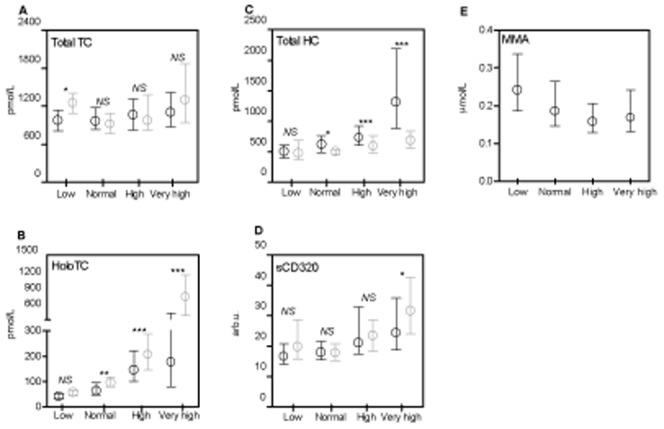
Medians and interquartile ranges of serum total transcobalamin(A), holo transcobalamin(B), total haptocorrin(C), sCD320(D), and methylmalonic acid(E). Black signature: patients not in Cbl therapy; grey signatures patients in Cbl therapy. Please note the split y-axis in figure 1B. NS: not significant (p>0.05), *:p<0.01, **:p<0.005, ***:p<0.0001, by comparing patients not in Cbl therapy to patients in Cbl therapy within same Cbl group using Mann-Whitney U-test. Abbreviations: Cbl: cobalamin, vitamin B12; TC: transcobalamin; holoTC: Cbl-saturated transcobalamin; HC: haptocorrin; sCD320: soluble transcobalamin receptor CD320; MMA: methylmalonic acid.

Blood samples were collected in test tubes without any additives, centrifuged at 2,300 g and analyzed for serum Cbl within 4 hours of sample collection. The surplus serum was removed and frozen at −20°C on the same day and stored until further analyzed.

We assessed the distribution of serum Cbl levels, age, and gender by withdrawing all Cbl measurements in the study period from the electronic laboratory information system.

### Biochemical Analyses

The serum samples were analyzed for total serum Cbl levels on Cobas 6000 E, Roche Diagnostics (www.roche.com) in the routine laboratory. All samples with Cbl levels >1476 pmol/L (upper scale limit of the routine assay) or unsaturated B12 binding capacity >6000 pmol/L (interferes with the routine assay [19]) were reanalyzed for total serum Cbl levels after diluting serum in 0.025 mmol/L sodium phosphate buffer containing 115 mmol/L NaCl and 1 g/L bovine albumin, pH 8.0 (Sigma Aldrich, St. Louis, MO, USA, catalog #: A7030, www.sigmaaldrich.com).

**Table 3:** Diagnostic associations to cobalamin levels.

				Groups according to serum cobalamin					
		<200 pmol/L^a^		200–600 pmol/L		601–1000 pmol/L		>1000 pmol/L	
**Diagnoses**		**n = 189**		**n = 190**		**n = 159**		**n = 94**	
**Alcoholism, n**		13		20		36		28	
Crude OR (95% CI)		1.00		1.59 (0.77–3.30)		3.96 (2.02–7.78)		5.74 (2.81–11.75)	
Adjusted^b^ OR (95% CI)		1.00		1.35 (0.64–2.82)		3.73 (1.88–7.39)		5.74 (2.76–11.96)	
**Liver diseases, n**		8		13		22		24	
Crude OR (95% CI)		1.00		1.66 (0.67–4.11)		3.63 (1.57–8.41)		7.76 (3.33–18.08)	
Adjusted^b^ OR (95% CI)		1.00		1.64 (0.66–4.09)		3.65 (1.57–8.50)		8.53 (3.59–20.23)	
**Cancer, n**		18		26		21		38	
Crude OR (95% CI)		1.00		1.51 (0.80–2.85)		1.45 (0.74–2.82)		6.45 (3.41–12.19)	
Adjusted^b^ OR (95% CI)		1.00		1.24 (0.64–2.39)		1.30 (0.66–2.58)		5.48 (2.85–10.55)	
**Renal diseases, n**		11		13		19		7	
Crude OR (95% CI)		1.00		1.19 (0.52–2.72)		2.20 (1.01–4.77)		1.30 (0.49–3.47)	
Adjusted^b^ OR (95% CI)		1.00		1.07 (0.46–2.48)		2.08 (0.95–4.56)		1.07 (0.40–2.88)	
**Autoimmune diseases, n**		15		12		12		9	
Crude OR (95% CI)		1.00		0.78 (0.36–1.72)		0.95 (0.43–2.09)		1.23 (0.52–2.92)	
Adjusted^b^ OR (95% CI)		1.00		0.86 (0.39–1.91)		1.00 (0.45–2.21)		1.28 (0.53–3.11)	
**Bronchopulmonary diseases, n**		24		31		31		25	
Crude OR (95% CI)		1.00		1.34 (0.75–2.38)		1.67 (0.93–2.98)		2.49 (1.33–4.66)	
Adjusted^b^ OR (95% CI)		1.00		1.16 (0.63–2.13)		1.58 (0.86–2.91)		1.89 (0.98–3.66)	
**Cardiovascular diseases, n**		35		37		31		26	
Crude OR (95% CI)		1.00		1.06 (0.64–1.78)		1.07 (0.62–1.82)		1.68 (0.94–3.01)	
Adjusted^b^ OR (95% CI)		1.00		0.82 (0.46–1.45)		0.93 (0.51–1.68)		1.14 (0.60–2.16)	
**Psychiatric diseases, n**		46		53		47		18	
Crude OR (95% CI)		1.00		1.20 (0.76–1.90)		1.30 (0.81–2.10)		0.74 (0.40–1.36)	
Adjusted^b^ OR (95% CI)		1.00		1.26 (0.78–2.02)		1.36 (0.84–2.22)		0.86 (0.46–1.62)	
**Diabetes mellitus type II, n**		29		22		13		12	
Crude OR (95% CI)		1.00		0.72 (0.40–1.31)		0.49 (0.25–0.98)		0.81 (0.39–1.66)	
Adjusted^b^ OR (95% CI)		1.00		0.58 (0.31–1.08)		0.42 (0.21–0.86)		0.61 (0.29–1.28)	
**Neurological diseases, n**		29		23		30		22	
Crude OR (95% CI)		1.00		0.76 (0.42–1.37)		1.28 (0.73–2.25)		1.69 (0.91–3.13)	
Adjusted^b^ OR (95% CI)		1.00		0.68 (0.37–1.23)		1.21 (0.68–2.13)		1.40 (0.74–2.64)	
**Gastrointestinal diseases, n**	50		47		52		24		
Crude OR (95% CI)	1.00		0.91 (0.58–1.45)		1.35 (0.85–2.15)		0.95 (0.54–1.68)		
Adjusted^b^ OR (95% CI)	1.00		1.02 (0.64–1.64)		1.46 (0.91–2.34)		1.12 (0.63–1.99)		

Diagnoses related to Cbl levels in groups in patients referred for measurement of serum Cbl levels and not in Cbl supplementation therapy (n = 632). Patients were allowed to have more than one diagnosis. Odds ratios and 95% confidence intervals were obtained by logistic regression analyses.

a Patients with Cbl levels ≤200 pmol/L were treated as reference group.

b Adjusted for age (reference age 56 years) and gender (female as reference). Abbreviations: Cbl: cobalamin, vitamin B12; OR: OR: Odds ratio; 95% CI: 95% confidence interval.

All blood samples were analyzed for serum concentrations of Cbl binding proteins, total transcobalamin (TC), Cbl-saturated TC (holoTC) and total HC according to the established in-house ELISA protocols [20]–[22]. We also analyzed all samples for the soluble form of the TC receptor, soluble CD320 (sCD320), according to our newly described ELISA protocol [23]. All serum samples from non-Cbl supplemented patients (see below) were analyzed for serum methylmalonic acid (MMA) levels at the Department of Clinical Biochemistry, Rigshospitalet, University of Copenhagen, Denmark. These analyses were performed with an optimized LC-MS/MS-version of the HILIC-based method [24] using a Waters Micromass LC-MS/MS system (www.waters.com). All samples from non-Cbl supplemented patients were also analyzed for serum creatinine on Cobas 6000 E, Roche Diagnostics (www.roche.com). From this, estimated glomerular filtration rate (eGFR) was calculated using the formula: 175× (serum creatinine (µmol/L)/88,4) ^−1,154^ × (age in years) ^−0,203^ × 0,742 (if patient is female), with no racial correction [25].

**Table 4:** Cobalamin related parameters in diagnoses.

		Cobalamin related parameters		
	Total TC, pmol/L	HoloTC, pmol/L	Total HC, pmol/L	sCD320, arb.u.
**Diagnoses**	**[600–1500]**	**[40–150]**	**[240–680]**	**[12–97]**
**Alcoholism, n = 97**	1070 (860–1290)	120 (61–240)	730 (590–1240)	22 (16–35)
**Liver diseases, n = 68**	1020 (850–1390)	130 (68–320)	740(540–1260)	25 (17–39)
**Cancer, n = 103**	1100 (890–1400)	77 (52–130)	770 (490–1420)	22 (17–32)
**Renal diseases, n = 50**	1230 (910–1470)	120 (54–225)	760 (550–980)	35 (23–45)
**Autoimmune diseases, n = 48**	1310 (890–1860)	110 (56–180)	690 (540–1070)	19 (16–27)
**Bronchopulmonary diseases, n = 111**	1060 (840–1380)	93 (49–160)	710 (540–1020)	20 (16–28)
**Cardiovascular diseases, n = 129**	1070 (850–1340)	80 (44–130)	650 (490–910)	20 (16–30)
**Psychiatric diseases, n = 164**	950 (820–1140)	65 (43–130)	660 (500–870)	18 (15–23)
**Diabetes mellitus type II, n = 76**	1100 (910–1340)	65 (43–110)	580 (470–810)	18 (16–25)
**Neurological diseases, n = 104**	990 (840–1200)	83 (47–160)	580 (470–880)	20 (15–30)
**Gastrointestinal diseases, n = 170**	940 (800–1190)	77 (44–170)	650 (510–800)	20 (16–30)

Median (interquartile ranges) [reference ranges] levels of Cbl related parameters divided according to diagnoses in patients referred for measurement of serum Cbl levels and not in Cbl supplementation therapy (n = 632). Abbreviations: Cbl: cobalamin, vitamin B12; TC: transcobalamin; holoTC: Cbl-saturated transcobalamin; HC: haptocorrin; sCD320: soluble transcobalamin receptor CD320.

All biochemical analyses were performed between March 2010 and January 2012.

### Clinical Data

Information on diagnoses of chronic or acute disease was obtained from the Electronic Patients Medical Chart at Aarhus University Hospital. All medical charts were examined by the same investigator. The data was collected according to the time of blood sampling. Hence, the diagnoses were related to the admission or outpatient procedure where the Cbl measurement was requested or, in case of prior chronic disease, diagnosed before the blood sampling. The diagnoses from the clinician(s) were considered valid without validation according to diagnostic criteria. Data on medical prescriptions were collected from the medical charts and from the Aarhus University Prescription Database [26]. Data collection was conducted between October 2010 and March 2011.

We analyzed disease patterns according to the defined Cbl level groups. Additionally, we analyzed levels of Cbl related parameters in the different diagnostic categories. This was done to examine whether specific alterations in the Cbl parameters was associated to a particular disease. In selecting the diagnostic categories we paid attention to diseases previously suggested to be associated with elevated serum Cbl and/or Cbl binding proteins (alcoholism, liver disease, myeloid diseases, lymphatic diseases, solid tumor cancer, renal disease and autoimmune diseases) [1]–[18]. Additionally, we added diagnoses of diabetes mellitus type II, cardiovascular (except patients with only hypertension), psychiatric, bronchopulmonary, neurologic, and gastrointestinal disease. If an organ-specific disease was malignant it was only included in the solid tumor cancer category. For simplicity, we pooled all malignant diagnoses (myeloid diseases, lymphatic diseases, and solid tumor cancer) into one category; cancer. Patients were allowed to have more than one diagnosis.

### Statistical Analyses

Statistical analyses were performed using GraphPad Prism® version 4.0 for Windows (GraphPad Software, San Diego, California, USA, www.graphpad.com), Stata® 11 (StataCorp LP, College Station, Texas, USA, www.stata.com), and Microsoft® Excel 2003 (Microsoft Corporation, Redmond, Washington, USA, www.microsoft.com).

Patients in either oral or parenteral Cbl replacement therapy were analyzed separately for Cbl related parameters and excluded from analyses of diagnostic associations to Cbl levels. 129 non-supplemented patients with eGFR <60 mL/min/1.73 m^2^ were excluded from analyses of MMA.

All data on Cbl related parameters were positively skewed and no suitable transformation was found. Thus, non-parametric statistical tests were used and levels of Cbl related parameters are reported as medians with interquartile ranges. Means for age were compared using one-way analysis of variance. Median levels of total TC, holoTC, total HC, sCD320 and MMA (MMA only for non-supplemented patients, see exclusion criteria) were compared by Kruskal-Wallis test. Mann-Whitney U-test was applied for testing the difference between Cbl-supplemented and non-supplemented patients for median levels of total TC, holoTC, total HC, and sCD320 levels. Correlations between the Cbl related parameters in non-supplemented patients were analyzed using Spearman’s rank correlation. Logistic regression analyses were performed to determine the associations between high Cbl levels and the selected diagnoses, using the group with low levels as reference. Results are presented as crude odds ratios (OR) and ORs adjusted for age (reference age 56 years) and gender (female as reference), with corresponding 95% confidence intervals (95% CI).

## Results

### Patient Characteristics

We collected blood samples from hospital-treated patients in order to analyze biochemical parameters and diagnostic patterns in patients with low (<200 pmol/L, normal (200–600 pmol/L), high (600–1000 pmol/L) and very high (>1000 pmol/L) Cbl levels. Samples from hospital-treated patients analyzed for Cbl during the study period (n = 12,070) showed a distribution amongst the four Cbl groups of 9% (low), 71% (normal), 13% (high) and 7% (very high). For comparison the distribution amongst Cbl requested from GPs (n = 27,977) was 11% (low), 77% (normal), 9% (high) and 3% (very high). The two populations were similar in age distribution, but the GP population covered more females (65% as compared to 56%).

We collected samples from a total of 825 patients. Six patients were excluded because of incomplete medical files. Notably, a 42-year old female patient with HIV (the only HIV positive patient) showed extremely high levels of Cbl binding proteins (total TC = 83,500 pmol/L, holoTC = 77,200 pmol/L and total HC = 23,700 pmol/L), but a normal sCD320 level (sCD320 = 22 arbitrary units (arb.u.)). HIV-infected patients have been suggested to have an altered Cbl metabolism, but reports show conflicting results [27]–[30]. This was not scrutinized further and the patient was excluded from further analyses of Cbl related parameters and diagnoses.

Table 1 displays the characteristics of the final study population of 818 patients. The mean age was 58.7 years (95% CI: 57.2–59.9, range: 18–99 years), and was significantly higher with higher Cbl levels. The gender distribution was 42% males and 58% females. The mean age of non-supplemented patients was 56.8 years (95% CI: 55.28–58.23, range 18–97 years).

A total of 22.7% of the patients (n = 186) were in Cbl supplementation therapy at the time of blood sampling. The distribution of Cbl supplemented patients across Cbl groups was: low: 5.5%, normal: 5.9%, high: 26.7%, very high: 52.8%. We were not able to elucidate why Cbl measurements were requested for these patients.

### Biochemical Markers

Table 2 shows the Cbl related parameters (total and holoTC, total HC, sCD320, and MMA) in non Cbl-supplemented patients. By Kruskal-Wallis test, we observed significantly higher levels of all five parameters with higher levels of Cbl (p<0.001). Median levels of total TC ranged from 970 to 1,100 pmol/L, holoTC from 41 to 180 pmol/L, total HC from 510 to 1,300 pmol/L, sCD320 from 17 to 24 arb.u., and MMA from 0.24 to 0.16 µmol/L across the four Cbl groups.

Among Cbl-supplemented patients the levels of Cbl related parameters also differed between the Cbl groups (Kruskal Wallis test: p<0.05), but we found a broader range in median holoTC from 57 to 720 pmol/L and a narrower range in median total HC from 480 to 680 pmol/L compared to the non Cbl-supplemented (table 2).

A total of 352 (56% of total) non Cbl-supplemented patients had levels above the reference intervals in one or more of the Cbl related parameters (total and holoTC, total HC, sCD320). This was unequally distributed among the four Cbl groups: low: 35 (19%), normal: 92 (48%), high: 131 (82%) and very high: 94 (100%).

Regarding MMA, 22% of the non Cbl-supplemented patients with normal kidney function had levels above the reference range (upper limit: 0.28 µmol/L) with the following distribution in the four Cbl groups; low: 57 (37%), normal: 36 (23%), high: 5 (4%), and very high Cbl: 12 (18%). Only 11 patients (2% of total) showed extremely high MMA levels >0.75 µmol/L (table 2).

We compared levels of Cbl related parameters between Cbl-supplemented and non-supplemented patients (figure 1). In the group with low Cbl levels, the levels of total TC were higher among supplemented patients (p<0.05). Total HC levels were higher among non-supplemented, especially in patients with high and very high Cbl levels (p<0.0001). In the group with very high Cbl levels, sCD320 levels were higher in supplemented patients (p<0.05).

Correlation analyses between the Cbl related parameters in non Cbl-supplemented patients showed positive correlations between Cbl levels and holoTC (Spearman’s rho: 0.74, 95% CI: 0.70–0.77) and total HC (Spearman’s rho: 0.60, 95% CI: 0.55–0.65). We found a moderately strong positive association between levels of sCD320 and holoTC, the soluble receptor and the receptor ligand (Spearman’s rho: 0.43, 95% CI: 0.36–0.49). MMA levels correlated negatively to total Cbl, holoTC and total HC levels (p<0.001).

We explored biochemical signs of impaired Cbl status in non-supplemented patients with normal kidney function and a plasma Cbl level >250 pmol/L (n = 312), the recommended decision level for ruling out Cbl deficiency [31]. Impaired Cbl status was defined as holoTC <40 pmol/L and/or MMA >0.28 µmol/L.Most of the patients meeting these criteria (n = 62) had Cbl levels between 250 and 600 pmol/L, but interestingly a relatively large group had Cbl levels >1000 pmol/L (table 2).

### Diagnostic Distribution

Table 3 shows the diagnostic categories divided according to cobalamin level groups. We found significant associations between higher Cbl levels and alcoholism, liver disease, and cancer, the latter only significant in patients with Cbl levels >1000 pmol/L. The risk of alcoholism and cancer was higher for males than for females (data not shown). The risk of renal disease in patients with Cbl levels 601–1000 pmol/L was elevated, but this association was attenuated by adjusting for age and gender. In bronchopulmonary disease, the higher risk among patients with Cbl levels >1000 pmol/L was also attenuated by adjustment for age and gender. For diabetes mellitus type II, a small protective effect of higher Cbl levels was seen, although only significant for patients with Cbl levels 601–1000 pmol/L. When stratifying the diagnostic category cancer into the three subtypes, myeloid diseases, lymphatic diseases, and solid tumor cancers, patients with Cbl levels >1000 pmol/L had higher risks of all three subtypes, with the highest risk for myeloid cancers. When stratifying liver diseases into alcohol and non-alcohol related, both types of liver disease were associated to high Cbl levels, with the highest risk estimates for alcoholic liver disease (table S1).

Table 4 shows the median levels of Cbl related parameters in the different disease categories. For four disease categories; alcoholism, liver, renal, and autoimmune diseases, median holoTC levels were high, although within reference range. Median total HC levels were above reference range in six categories, and especially high in cancer and renal disease patients. Total TC and sCD320 levels showed no specific disease association, although the latter was found higher in renal disease than in any of the other disease categories.

## Discussion

This study was conducted to assess the possible clinical implications of elevated serum Cbl levels observed in hospital–treated patients referred for measurement of Cbl. In addition, we wanted to study Cbl levels and diagnoses in relation to the Cbl binding proteins, the soluble TC receptor, sCD320, and MMA.

When interpreting the results several issues must be taken into account. First, our focus was an examination of patients referred for analysis of Cbl, and thus the study do not necessarily represent the true distribution of patients with high Cbl levels. Nor does it allow us to conclude whether high Cbl levels can be used as a biomarker for specific diseases. Third, in order to focus on patients with high Cbl levels, this group, especially those with very high levels, was oversampled compared to the actual prevalence of patients with high Cbl levels in the group of patients referred to Cbl measurement. Fourth, the age of the patients was higher with higher Cbl levels. It is expected that older patients have more morbidities, and the disease associations might be related to the higher age, although the adjusted estimates did not alter the associations substantially. But since our underlying motive was to understand the unexpected high Cbl levels in patients suspected of Cbl deficiency, these issues are judged to be of minor importance for the interpretation of the results.

We used the local reference interval to make cut-offs for the Cbl level groups, thus defining high Cbl levels as above the upper reference limit. Reference intervals vary with the applied measurement methods and the relevant populations, so the exact cut-offs can not readily be applied in other settings.

Our comparison of the diagnostic patterns in patients with different Cbl levels showed several interesting features. We found that high Cbl levels were significantly associated to alcoholism and liver disease. This is consistent with earlier results [2]; [10]; [15]. In these diseases, a dominant finding in our study was a high level of HC.

Previous studies have described the association between high Cbl levels and different malignancies, possibly also related to high HC levels [3]–[7]; [9]; [11]–[12]; [16]; [18]. Our results show that cancer is significantly associated to high Cbl levels, and these cancer patients had high HC levels Although, we found only a small number of cases in each group, all three subgroups, myeloid, lymphatic, and solid tumors were associated to Cbl levels >1000 pmol/L.

Previously, renal disease has been reported associated to high Cbl levels and concurrent high holoTC and saturated HC levels [1]. In part, we confirmed such an association and showed that the high Cbl levels were caused by high levels of total HC and holoTC levels in the upper end of the reference range.

We report a novel (though not uniformly observed) association between bronchopulmonary diseases and high levels of Cbl. The high Cbl levels were associated to high HC levels. Earlier studies from our lab showed HC production in the bronchial mucosa [32], but whether this is related to the high circulating levels of HC in bronchopulmonary disease remains unresolved.

As mentioned above our results do not allow us to conclude whether high Cbl could be a biomarker for specific diseases. But our findings suggest further studies to clarify this issue. In particular, the high prevalence of cancer in our study population spurs to further study Cbl metabolism in cancer.

We excluded patients who were requested for Cbl measurement by their GPs, because clinical data and medical charts from these are not available for research purposes. Our crude analysis of all Cbl measurements performed during the study period showed that low levels of Cbl were more frequent (11% as compared to 9%) while very high levels were less frequent (3% as compared to 7%) in the GP population than in the hospital-treated population. While the age distribution was similar, the distribution between men and women differed. Taken together these data suggests that our results can not directly be transferred to GP-treated patients, but that the problem of unexpected high levels of Cbl may also exist for GP-treated patients.

Previous studies have compared Cbl levels to the concentration of other Cbl related parameters [33]. However, only few studies include measurement of both total TC, holoTC and total HC, and to our knowledge this study is the first to include measurement of sCD320. We found that Cbl levels >600 pmol/L were related to high total HC levels rather than high total TC. Further studies are needed to clarify the disease specific associations to high HC levels.

Our data allowed us to examine the potential relations between the novel biomarker, sCD320, and other Cbl related parameters. While sCD320 correlated positively to the Cbl binding proteins, MMA and sCD320 levels did not correlate, suggesting that sCD320 levels are not influenced by cellular Cbl status. Interestingly, we found the strongest correlation between sCD320 and the receptor ligand, holoTC. This suggests that the metabolically active fraction, and possibly receptor activity, could influence receptor release from the cell surface and into the circulation. Further supporting this hypothesis, we found the highest median sCD320 levels in the Cbl supplemented patients with Cbl above 1000 pmol/L and extremely high holoTC levels. The possible underlying mechanisms demand further substantiation.

Finally, we explored the relation between sCD320 and diagnostic patterns. Earlier studies have shown increased receptor activity in proliferating cells and a possible cell-cycle regulation of the receptor gene expression [34]–[36]. Hence, diseases where cell proliferation is evident, e.g. cancer, infectious and inflammatory diseases, could be a reasonable suggestion for a possible association. We did not find any obvious clinical associations to sCD320 levels, nor did we find evidence to suggest sCD320 as a novel biomarker for Cbl deficiency.

In conclusion, our study strongly suggests that high Cbl levels in patients referred for measurement of Cbl should be taken into account in the diagnostic procedure as it may indicate underlying disease. Notably, alcoholism, liver disease and cancer were associated to high Cbl levels, and all of these diseases were associated to high HC levels.

## Supporting Information

Table S1 **Diagnostic associations to cobalamin levels, subgroup diagnoses.** Diagnosis subgroups related to Cbl levels in groups in patients referred for measurement of serum Cbl levels and not in Cbl supplementation therapy (n = 632). Patients were allowed to have more than one diagnosis. Cancer was divided into three groups: myeloid, lymphatic and solid tumors. Liver disease was divided into alcoholic and other liver diseases. Odds ratios (OR) and 95% confidence intervals (CI) were obtained by logistic regression analyses. ^a^Patients with Cbl levels ≤200 pmol/L were treated as reference group. ^b^Adjusted for age (reference age 56 years) and gender (female as reference). Abbreviations: Cbl: cobalamin, vitamin B12; OR: OR: Odds ratio; 95% CI: 95% confidence interval.
(DOC)
