# Supplementary material for: Cobalamin Related Parameters and Disease Patterns in Patients with Increased Serum Cobalamin Levels
Source: PLoS One. 2012 Sep 21;7(9):e45979. doi: 10.1371/journal.pone.0045979 (PMC3448722; doi:10.1371/journal.pone.0045979)
Supplement: Table S1 — Diagnostic associations to cobalamin levels, subgroup diagnoses. Diagnosis subgroups related to Cbl levels in groups in patients referred for measurement of serum Cbl levels and not in Cbl supplementation therapy (n = 632). Patients were allowed to have more than one diagnosis. Cancer was divided into three groups: myeloid, lymphatic and solid tumors. Liver disease was divided into alcoholic and other liver diseases. Odds ratios (OR) and 95% confidence intervals (CI) were obtained by logistic regression analyses. aPatients with Cbl levels ≤200 pmol/L were treated as reference group. bAdjusted for age (reference age 56 years) and gender (female as reference). Abbreviations: Cbl: cobalamin, vitamin B12; OR: OR: Odds ratio; 95% CI: 95% confidence interval. (DOC) [file pone.0045979.s001.doc]

**Table S1.**

|  |  | **Groups according to serum cobalamin** | |  |
| --- | --- | --- | --- | --- |
|  | **< 200 pmol/L^a^** | **200-600 pmol/L** | **601-1000 pmol/L** | > **1000 pmol/L** |
| **Diagnoses** | **n = 189** | **n = 190** | **n=159** | **n = 94** |
| **Alcoholic liver disease, n** | 2 | 4 | 15 | 13 |
| Crude OR (95 % CI) | 1.00 | 2.01 (0.36;11.11) | 9.74 (2.19;43.27) | 15.01 (3.31;68.02) |
| Adjusted^b^ OR (95 % CI) | 1.00 | 1.85 (0.33;10.31) | 9.39 (2.11;41.90) | 15.37 (3.35;70.57) |
| **Other liver diseases, n** | 6 | 9 | 7 | 11 |
| Crude OR (95 % CI) | 1.00 | 1.52 (0.53;4.35) | 1.40 (0.46;4.27) | 4.04 (1.45;11.30) |
| Adjusted^b^ OR (95 % CI) | 1.00 | 1.61 (0.55;4.69) | 1.47 (0.48;4.49) | 4.64 (1.62;13.30) |
| **Myeloid diseases, n** | 1 | 5 | 5 | 9 |
| Crude OR (95 % CI) | 1.00 | 5.08 (0.59;43.91) | 6.10 (0.71;52.80) | 19.91 (2.48;159.62) |
| Adjusted^b^ OR (95 % CI) | 1.00 | 4.31 (0.49;37.55) | 5.58 (0.64;48.46) | 18.05 (2.23;145.95) |
| **Lymphatic diseases, n** | 6 | 14 | 3 | 12 |
| Crude OR (95 % CI) | 1.00 | 2.43 (0.91;6.45) | 0.59 (0.14;2.38) | 4.46 (1.62;12.30) |
| Adjusted^b^ OR (95 % CI) | 1.00 | 2.31 (0.86;6.21) | 0.57 (0.14;2.32) | 4.24 (1.52;11.85) |
| **Solid tumor cancer, n** | 11 | 7 | 14 | 19 |
| Crude OR (95 % CI) | 1.00 | 0.62 (0.23;1.63) | 1.56 (0.69;3.55) | 4.10 (1.86;9.03) |
| Adjusted^b^ OR (95 % CI) | 1.00 | 0.46 (0.19;1.25) | 1.38 (0.59;3.24) | 3.16 (1.39;7.20) |
